# Spectrum-Wide Exploration of Human Adenoviruses for Breast Cancer Therapy

**DOI:** 10.3390/cancers12061403

**Published:** 2020-05-29

**Authors:** Nicolas Mach, Jian Gao, Lukas Schaffarczyk, Sebastian Janz, Eric Ehrke-Schulz, Thomas Dittmar, Anja Ehrhardt, Wenli Zhang

**Affiliations:** 1 Virology and Microbiology, Center for Biomedical Education and Research (ZBAF), Department of Human Medicine, Faculty of Health, Witten/Herdecke University, 58453 Witten, Germany; Nicolas.Mach@uni-wh.de (N.M.); Jian.Gao@uni-wh.de (J.G.); Lukas.Schaffarczyk@uni-wh.de (L.S.); Sebastian.Janz@uni-wh.de (S.J.); Eric.Ehrke-Schulz@uni-wh.de (E.E.-S.); 2 Institute of Immunology, Center for Biomedical Education and Research (ZBAF), Department of Human Medicine, Faculty of Health, Witten/Herdecke University, 58453 Witten, Germany; Thomas.Dittmar@uni-wh.de

**Keywords:** adenovirus, adenovirus receptors, breast cancer, broad spectrum, cellular entry, high-throughput screening, virotherapy

## Abstract

Oncolytic adenoviruses (Ads) are promising tools for cancer therapeutics. However, most Ad-based therapies utilize Ad type 5 (Ad5), which displays unsatisfying efficiency in clinical trials, partly due to the low expression levels of its primary coxsackievirus and adenovirus receptor (CAR) on tumor cells. Since the efficacy of virotherapy strongly relies on efficient transduction of targeted tumor cells, initial screening of a broad range of viral agents to identify the most effective vehicles is essential. Using a novel Ad library consisting of numerous human Ads representing known Ad species, we evaluated the transduction efficiencies in four breast cancer (BC) cell lines. For each cell line over 20 Ad types were screened in a high-throughput manner based on reporter assays. Ad types featuring high transduction efficiencies were further investigated with respect to the percentage of transgene-positive cells and efficiencies of cellular entry in individual cell lines. Additionally, oncolytic assay was performed to test tumor cell lysis efficacy of selected Ad types. We found that all analyzed BC cell lines show low expression levels of CAR, while alternative receptors such as CD46, DSG-2, and integrins were also detected. We identified Ad3, Ad35, Ad37, and Ad52 as potential candidates for BC virotherapy.

## 1. Introduction

Cancer is the second leading cause of death in industrialized countries and constitutes an enormous burden on the health-care system. Among all cancer types, breast cancer (BC) is the most commonly diagnosed cancer (24.2%) and the leading cause of cancer deaths (11.6%) in women worldwide [1]. Treatment options of BC patients also depend on the grading and therefore, specific features of the BC type. Hormone receptor-positive (estrogen receptors, ER+; progesterone receptors, PR+) BC can be treated with hormone-blocking agents comprising selective estrogen receptor modulators or aromatase inhibitors. Estrogen receptor-negative (ER−) BC are primarily treated with radiochemotherapy targeting fast-replicating cancer cells [2]. However, chemotherapy also causes damage to physiologic, fast-growing cells. Since 25–30% of BCs overexpress the human epidermal growth factor receptor 2 (HER2), monoclonal antibodies such as trastuzumab and pertuzumab against HER2 have been developed to target the HER2 protein. These monoclonal antibodies prevent growth factors from binding and stimulating this receptor, thus, effectively blocking the growth of the cancer cells [3,4]. Triple-negative breast cancers (TNBCs) are characterized by a negative profile for all three markers above and account for 10–15% of all BCs. Furthermore, TNBCs usually display a more aggressive growth pattern and are associated with a poorer prognosis than other BC types. TNBC is typically treated with a combination of primary surgery, radiation therapy, and adjuvant chemotherapy. Despite this treatment regimen, TNBCs are associated with a high mortality [5,6], highlighting the need for further research in this patient group.

As a novel therapeutic concept, oncolytic virotherapy lately has attracted considerable attention [7,8,9]. Virotherapeutics are based on two basic concepts: delivery of therapeutic genes to target tumor cells and tumor-selective replication of oncolytic viruses, which results in tumor cell lysis [10,11,12]. Virotherapeutics originated from clinical reports more than 100 years ago already demonstrated that cancer regression was coincidental with simultaneously occurring viral infections. Based on this observation, body fluids that contained human or animal viruses were used to transmit infections to patients with cancer in early clinical trials [13,14]. To improve the therapeutic effect, the characteristics of oncolytic viruses need to be optimized to achieve high efficacy and tumor specificity. During the past 30 years, tropism determinants of many different virus families have been identified and characterized. Most importantly, modern genetic engineering systems have been developed for almost every virus family, allowing the generation of viruses with improved oncolytic properties [15,16,17]. Finally, our understanding of cancer has also improved with the availability of diagnostic markers and detailed human genome information [2,18,19,20]. Although genetically modified viruses can also be explored to treat noncancerous diseases, cancer is the most often targeted disease as evidenced from the worldwide clinical trials database. Different viruses such as adenovirus (Ad), vaccinia virus, and herpes simplex virus have been evaluated in clinical setting to treat tumor patients.

Ads are medium-sized (70–100 nm in diameter), nonenveloped icosahedral viruses composed of a nucleocapsid and a double-stranded linear DNA genome with an average length between 27 and 36 kb. In humans more than 100 Ad types have been identified which can be divided into 7 genetically diverse species (A–G). Although human Ads cause significant numbers of respiratory, ocular, and gastrointestinal diseases, incidences of severe diseases caused by Ads only occur in immunocompromised individuals. Among the general population Ad infections are usually resolved quickly and result in life long immunity to the virus [21].

Since its first conversion from wild-type virus to a gene delivery vector in the beginning of 1990s [22], Ads have been recognized as the most efficient vehicles to deliver genes in vivo and as highly efficient tumor-killing (oncolytic) agents. Predominantly, oncolytic vectors derived from Ad type 5 were used in clinical trials. Although Ads can effectively transfer genes in vitro and in vivo, various limitations of the commonly used Ad5 exist. For instance, efficacy is frequently hampered by the high rates of neutralizing immunity, estimated as high as 90% in some populations, that promote vector clearance and limit bioavailability for tumor targeting following systemic delivery [23,24]. Active tumor targeting is also hampered by the ubiquitous nature of the Ad5 receptor, which is the human coxsackievirus and Ad receptor (CAR). As shown in previous studies, CAR expression levels were comparably low or were not detectable on many cancer cell lines, including the commonly used BC cell line MCF-7 [25,26,27].

In this study, we compared over 20 Ad types representing species B1, B2, C, D, E, and G regarding their ability to transduce human BC cell lines and breast epithelia cells. Among these Ad types, Ad5 had the highest transduction efficiency in breast epithelia cells. However, this was not true for BC cell lines. Based on this finding, we further compared the oncolytic efficacy of the selected Ad types to Ad5. Here, we identified several novel Ad candidates showing considerably higher transduction rates than Ad5 in BC cells. Analysis of major Ad receptor expression levels on BC cell lines correlated with current knowledge of Ad receptor utilization by respective Ads and therefore confirmed our findings. To our knowledge, this is the first study evaluating a broad spectrum of human Ad types in a panel of human BC cell lines and breast epithelia cells for BC targeting. The results of this study have important implications for optimized Ad-based BC virotherapy.

## 2. Results

**Figure 1:**
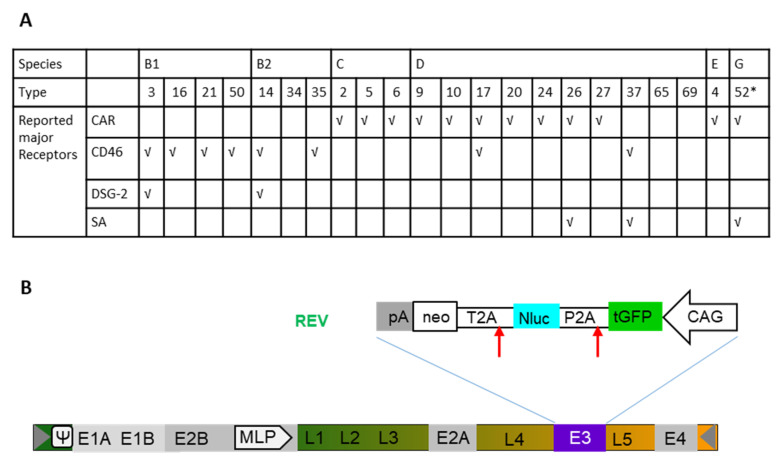
Reporter-labelled human adenoviruses (Ads). (**A**) Overall, 22 adenovirus types (Ad type number) from 6 species were used in transduction of breast cancer (BC)-related cells. The previously reported receptor usage is indicated (√). (**B**) Construction of human Ad expressing turbo green fluorescent protein (tGFP), nanoluciferase (Nluc), and neomycin resistance (neo) under the control of the hybrid construct consisting of the cytomegalovirus (CMV) enhancer fused to the chicken beta-actin promoter (CAG) This cassette was inserted in the E3 genomic region. *, it is of note that Ad52 used here is only labelled with turbo GFP (tGFP)-neo. Therefore, Ad52 is not presented in luciferase-based assays.

Twenty-two Ad types representing six Ad species were utilized in this study (Figure 1A). As previously described [28], these human Ads contain an expression cassette including turbo green fluorescent protein (tGFP), nanoluciferase (Nluc), and neomycin resistance (neo) located in the early gene region E3 in the reversed direction to the adenoviral genome (Figure 1B). For each experiment, the commonly used vector type Ad5 was applied as control. In the present study, we wanted to address the question which Ad types can efficiently transduce human BC cell lines, while only modestly transducing the breast epithelial cell line M13SV1. Furthermore, we investigated whether transduction efficiencies of Ads correlate with specific features of the BC type such as grading and receptor expression and which Ad types induce efficient tumor cell lysis. To answer these questions, we measured the transduction efficiency via a high-throughput screening based on luciferase assays, quantified GFP-positive cells using flow cytometry, investigated vector genome cell entry, and analyzed expression levels of Ad receptors and integrin coreceptor on each BC cell line. Finally, in a translational approach, we performed oncolytic assay to investigate the therapeutic potential of selected Ad types within the scope of breast cancer virotherapy.

### 2.1. High-Throughput Screening of a Panel of Ads in Human BC Cell Lines and Breast Epithelial Cell Line M13SV1

**Figure 2:**
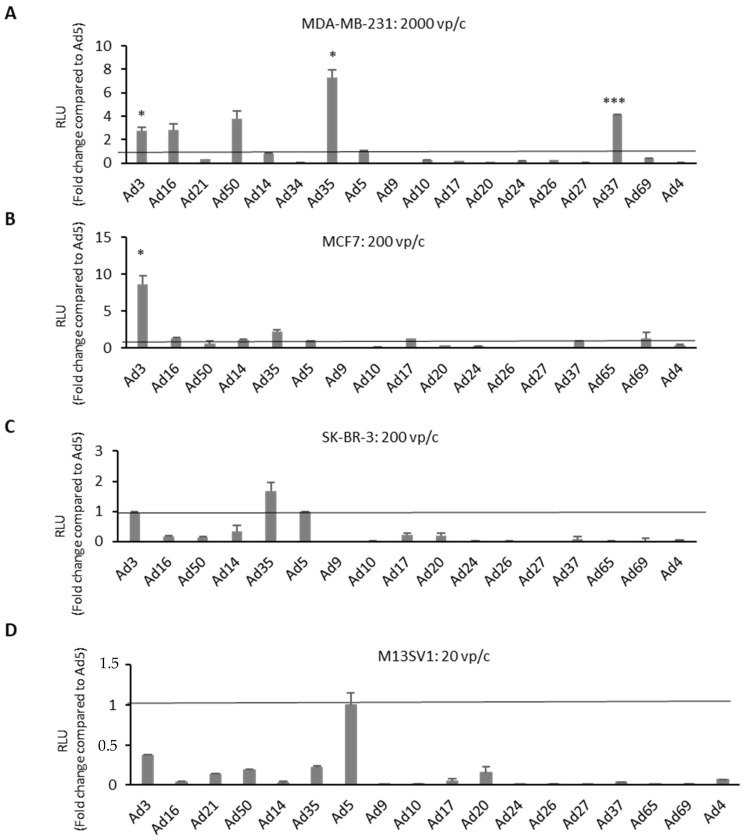
High-throughput screening of the reporter-tagged human adenovirus library in human BC-related cells. Transgene expression levels of different adenovirus types (Ad type number) were evaluated in BC-related cell lines. Cells were infected with each virus at various virus particle numbers per cell (vp/c). Shown are (**A**) MDA-MB-231 cells at 2000 vp/c, (**B**) MCF7 cells at 200 vp/c, (**C**) SK-BR-3 cells at 200 vp/c, and (**D**) breast epithelia cells M13SV1 infected with at 20 vp/c is used as control. Results from other vp/c are shown in the Figure S1. Luciferase expression levels were measured 24 h postinfection by addition of furimazine substrate and expressed as relative light units (RLU). Levels were compared to the commonly used adenovirus type 5 (Ad5) and indicated as fold change. Error bars, ±SEM of three independent experiments. * *p* < 0.05; *** *p* < 0.001; compared to Ad5 control.

In a preceding study [28], Hs 578T BC cells were screened by applying the Ad library at different viral particle numbers per cell (vp/c) and measuring the transgene expression levels via luciferase assays. Ad3-, Ad16-, Ad50-, Ad35-, and Ad37-infected cells exhibited significantly higher luciferase expression levels if directly compared to controls infected with Ad5. In the current study, three additional breast BC cell lines (SK-BR-3, MCF-7, and MDA-MB231) and a breast epithelial cell line (M13SV1) were included to investigate the oncolytic potential of Ad vectors. BC cell lines and breast epithelial cells were infected with a broad range of virus dosages (20, 200, 2000, and 20,000 vp/c). After 24 h of incubation, nanoluciferase expression levels were measured via luciferase assays (Figure 2 and Figure S1). In all BC cell lines, there were always at least one or more Ad types exhibiting higher luciferase expression levels compared to Ad5.

Interestingly, analyses of luciferase expression levels in another TNBC cell line (MDA-MB-231) revealed a similar trend as observed in Hs 578T cells, which was not the case in the other two analyzed BC cell lines, MCF7 and SK-BR-3. Ad3-infected MCF7 cells demonstrated an eightfold increased luciferase level compared to Ad5. All species B Ads and a few species D Ads (Ad17, Ad37, and Ad69) showed comparable or slightly higher luciferase expression levels than Ad5. However, in SK-BR-3 cells, only Ad3- and Ad35-infected cells revealed comparable or modestly higher luciferase expression levels than Ad5. In contrast to the results obtained in BC cell lines, Ad5 demonstrated the highest transduction efficiency among all tested Ad types in the breast epithelial cells M13SV1.

### 2.2. Quantification of Transgene-Positive Cells

**Figure 3:**
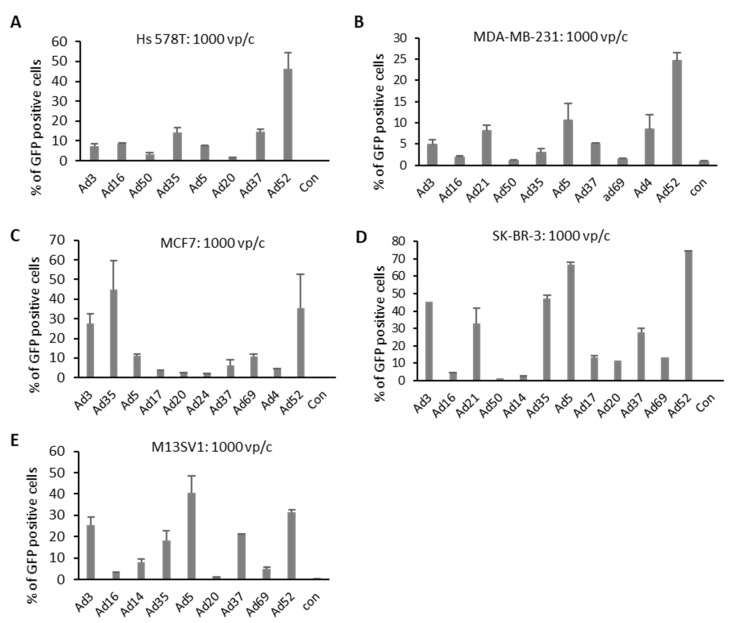
Number of GFP-positive cells after virus infection. Cells were infected with 10 Ads at 1000 viral particle per cell (vp/c), and GFP expression levels were analyzed 24 h postinfection by flow cytometry analyses. Uninfected cells (negative controls) were used to set the background gate below 1%. Percentage provided indicates percent of GFP-positive cells. A total of 10,000 viable cells were counted. (**A**–**D**) BC-originated tumor cell lines. (**E**) Breast epithelia cells M13SV1 are used as control. Error bars represent mean ± SD (*n* = 2).

High-throughput screening of Ads highlighted several Ad types potentially suitable for enhanced BC targeting. To further explore these selected Ads, BC cell lines were infected with respective Ads and the percentage of transgene-positive cells was quantified. Selected Ad types were applied to the four BC cell lines and one breast epithelial cell line (M13SV1) using 1000 vp/c. GFP expression was measured via flow cytometry 24 h postinfection and representative pictures of infected cells were collected (Figure 3 and Figures S2–S4). In both TNBCs, Hs 578T and MDA-MB-231, species G virus Ad52 revealed a significantly higher percentage of GFP-positive cells than Ad5. In MCF7 cells, infected with Ad3, Ad35, and Ad52, revealed a higher percentage of GFP-positive cells than those transduced with Ad5. However, in SK-BR-3 cells, 70% of Ad5-infected cells were positive for GFP expression. Other Ad types exhibited either a similar (Ad52) or slightly lower GFP expression (Ad3, Ad21, Ad35, and Ad37) than Ad5. In concordance with the results obtained in luciferase expression measurements, Ad5 again resulted in the highest level of GFP-positive cells among all analyzed Ad types in M13SV1 cells.

### 2.3. Cellular Entry of Ads 3 h after Infection

**Figure 4:**
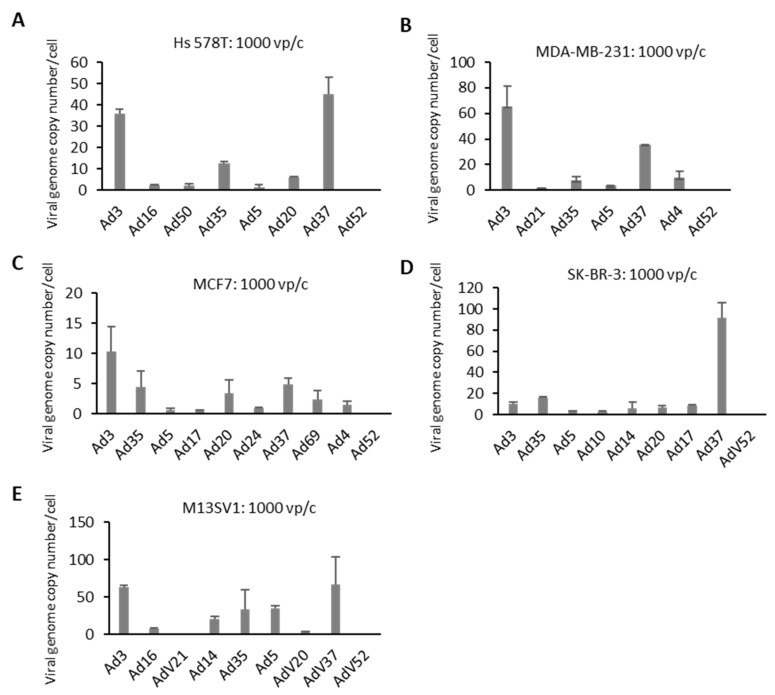
Virus internalization efficiency in BC cell lines. Cells were infected with individual viruses at 1000 viral particles per cell (vp/c) for 3 h to quantify internalized viral genome copy numbers (VCN), which were quantified by quantitative real-time PCR (qPCR) and expressed as VCN per cell. (**A**–**D**) BC-originated tumor cell lines. (**E**) Breast epithelia cells M13SV1 are used as control. Error bars represent mean ± SD (*n* = 2).

In the next step, the cellular entry of selected Ad types was evaluated. Cells were infected with 1000 vp/c. Briefly, 3 h postinfection, cells were washed and collected to isolate total DNA for quantification of virus genome copy numbers using quantitative PCR (Figure 4). TNBC cell lines, Hs 578T and MDA-MB-231, showed a similar trend concerning the amount of internalized virus genome copy numbers. In both cell lines, Ad3 and Ad37 demonstrated significantly higher infection rates compared to Ad 5 at 3 h postinfection. In MCF7 cells, Ad3 displayed the highest infection rates, followed by Ad37, Ad35, and Ad20. SK-BR-3 cells infected with Ad37 revealed the highest efficiency with respect to genome uptake. Other species B and D Ads also demonstrated a greater amount of intracellular adenoviral genome copies compared to Ad5. When analyzing M13SV1 control cells, the tested Ad types showed comparable (Ad14 and Ad35) or slightly higher (Ad3 and Ad37) genome entry efficiencies than Ad5.

### 2.4. Ad Receptor Expression Levels

**Figure 5:**
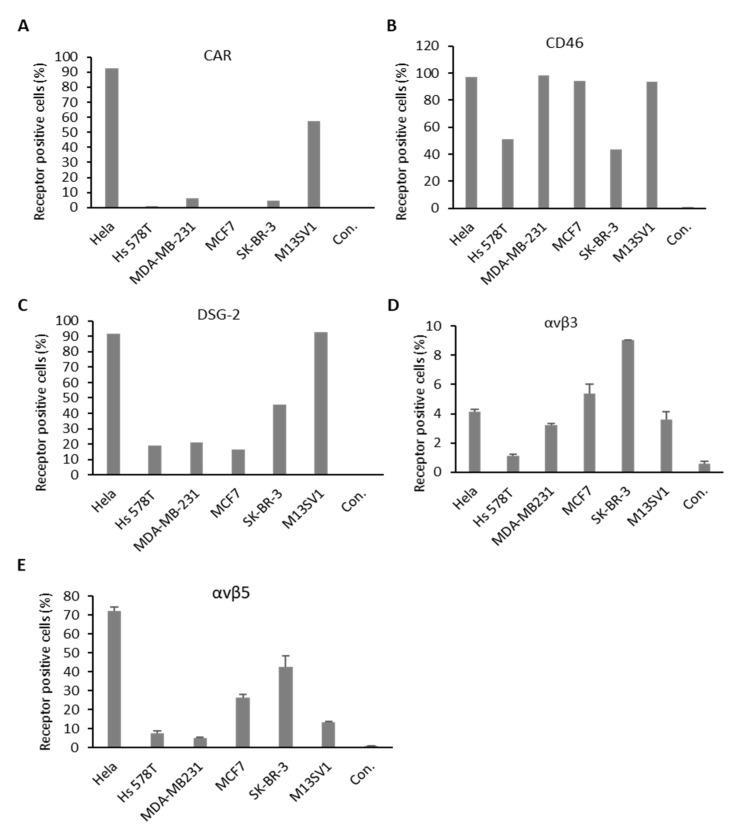
Major adenovirus receptor expression levels on BC cell lines. Cells were stained with antibodies against coxsackievirus and adenovirus receptor (CAR) (**A**), cluster of differentiation 46 (CD46) (**B**), desmoglein-2 receptor (DSG-2) (**C**), and integrins αvβ3 and αvβ5 (**D,E**). Receptor expression was measured via flow cytometry, expressed as percentage of CAR, CD46, DSG-2, and integrin-receptor-positive cells. Hela cells were used as positive control. Unlabeled cells (negative controls) were used to set the background gate below 1%. A total of 10,000 viable cells were counted.

Several major receptors used by different Ad types during the process of infection were identified in the past (Figure 1A). To understand the mechanisms behind cellular infection and transduction of Ads utilized in this study, the expression levels of major Ad receptors and integrin coreceptors were also examined for each cell line. Hela cells served as positive control in this experiment (Figure 5 and Figures S5 and S6). All examined BC cell lines exhibited relatively low or even no CAR expression, whereas the control cell line (M13SV1) displayed high levels of CAR expression. Concerning the expression of the CD46 receptor, tested cell lines demonstrated a high quantity of CD46-positive cells. Roughly, 50% of Hs 578T and SK-BR-3 cell were CD46 positive. However, analyses of MDA-MB-231, MCF7, and control cells (M13SV1) revealed that almost 100% of these cells were CD46 positive. The proportion of DSG-2 receptor-positive cells was 100% for the M13SV1 cell line, 50% in the SK-BR-3 cell line, and approximately, 20% in the cell lines Hs 578T, MDA-MB-231, and MCF7. With respect to coreceptors, we examined the most often studied integrins, αvβ3 and αvβ5. SK-BR-3 demonstrated the highest expression of both receptors among all tested BC-related cell lines. However, the αvβ3 integrin expression in all cell lines was relatively low compared to the other receptor types analyzed in this study (below 10%). Both TNBCs displayed lower number of positive cells compared to the other analyzed cell lines. It is of note that the integrin expression levels in the control cell line (M13SV1) were not higher than in the MCF7 and SK-BR-3 cell lines.

### 2.5. Oncolytic Potential of Ad Types in BC Cells

**Figure 6:**
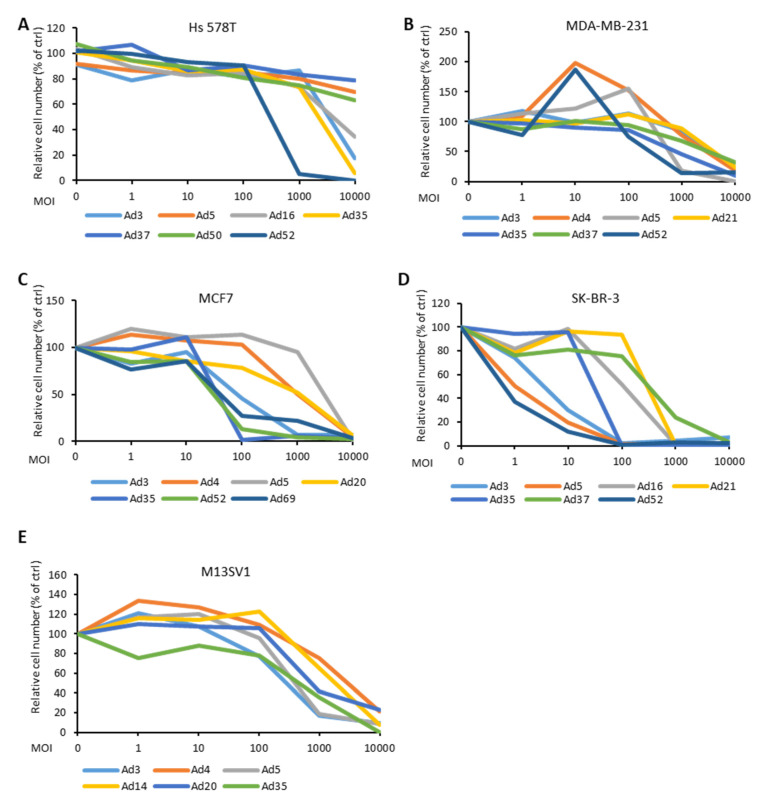
Cytotoxicity in response to infection with the different Ad types at various multiplicity of infection (MOI) (0, 1, 10, 100, 1000, and 10,000 virus particles per cell (vp/c)). Cell cytotoxicity was assessed by crystal violet staining (Figure S7) and then furthermore, quantified by measuring the absorbance of solubilized crystal violet dye at 570 nm. The data is displayed as percentage (%) of viable cells (i.e., stained cells) exposed to infection in relation to viable cells in the control (ctrl, uninfected) sample.

Efficient cell entry and transduction can represent predetermining factors enhancing the therapeutic effect of virotherapy. However, whether the viruses that enter the cells and express their genes may, meanwhile, induce tumor cell lysis is still unclear. We infected each BC cell line and the control cell line M13SV1 with selected Ad types, which demonstrated efficient transduction and infection rates in experiments described in previous sections. Ads were applied in a 10-fold dilution manner with MOIs ranging from 10,000 to 1. During a time frame of 1–2 weeks, cell lysis was observed, and the remaining cells were stained to visualize the lytic effect of the viruses (Figure 6 and Figure S7). As shown, Ad52 exhibited the most distinctive oncolytic effect in Hs 578T cells, while the MDA-MB-231 cells were lysed by Ad5, Ad35, and Ad52 to a comparable degree. In MCF7 cells, Ad3, Ad35, Ad52, and Ad69 demonstrated a 100-fold higher potency to lyse BC cells compared to Ad5. Ad3, Ad5, and Ad52 lysed SK-BR-3 cells with a similar high efficiency. Ad3, Ad20, and Ad52 showed a comparable or slightly lower lytic effect compared to that of Ad5 in the M13SV1 cells which served as control.

## 3. Discussion

The primary goal of this study was to identify alternative Ad types for enhanced BC virotherapy. To achieve efficient Ad-based therapy for cancer, the very first aspect to consider is the cellular entry of Ad, which is dependent on the expression levels of the primary receptors on the targeted tumor cells. Furthermore, virus-derived transgene expression levels in targeted tumor cells also represent an essential factor when considering the insertion of tumor suppressing or immunomodulatory genes into the vector’s genome. However, the most important parameter is the vector’s potency to cause cell lysis in tumor tissue, which partly depends on transgene expression and the virus replication efficiency in the targeted tumor cells.

In this study, the screening of a broad range of over 20 human Ad types was performed with four BC cell lines (Hs 578T, MCF-7, SK-BR-3, and MDA-MB231) and one breast epithelial cell line (M13SV1). A luciferase and GFP reporter gene expression cassette, which was previously cloned into the deleted E3 region of the applied Ad genomes enabled the rapid screening of Ads in a high-throughput manner [28]. In concordance to our previous observation regarding one TNBC cell line (Hs 578T) [28], in the current study, one or more Ad types showed higher luciferase expression levels than Ad5 in each BC cell line. In contrast to this observation, M13SV1 cells infected with Ad5 indicated highest luciferase expression levels correlating with efficient uptake and robust vector-derived transgene expression levels in this cell line. Based on this observation, we further filtered the broad range of utilized Ad types by selecting the Ads with the highest transgene expression levels. The identified 10 Ad types were additionally analyzed regarding their GFP expression utilizing flow cytometry in BC and breast epithelial cell lines. Notably, the results of the GFP measurements exhibited a similar trend compared to the previous results based on luciferase experiments, but this study also identified other BC-cell-line-specific Ad types, which may be converted into cancer-specific oncolytic Ads. Note that some discrepancies regarding luciferase expression levels and the percentage of GFP-positive and, therefore, transduced cells for single viruses were observed. However, one parameter quantifies total transgene expression levels in a population of cells and the other parameter measures the amount of transduced cells independent of transgene expression. GFP expression in a population of cells was also analyzed and we observed a similar trend with respect to transgene expression levels compared to luciferase expression levels. It has to be emphasized that not all Ad types were evaluated using all types of assays.

Besides vector-derived transgene expression levels, receptor-dependent infection efficiency is another important aspect determining the potency of an oncolytic Ad. CAR was the first Ad receptor identified and serves as a primary functional receptor for Ad5. However, it has been previously demonstrated that the therapeutic effect of Ad5 is restricted to the low CAR receptor expression levels on the targeted tumor cells [25,26,27]. To improve the therapeutic efficacy of adenoviral vectors, one promising approach is to introduce fiber modifications by replacing the fiber knob from Ad5 with a knob from an alternative Ad type [25]. Here, we explored the broad spectrum of alternative Ad types as an alternative strategy. Ads vary greatly in their tissue tropism and pathologies due to their utilization of different receptors during infection. Besides CAR, other major Ad receptors, such as the cluster of differentiation 46 (CD46), the desmoglein-2 receptor (DSG-2), and sialic acid (SA), have been identified. Most species B and some species D human Ads were confirmed to use the ubiquitously expressed membrane protein CD46 as a primary cellular entry receptor [29,30]. DSG-2 is utilized by Ad3, Ad7, Ad11, and Ad14 as major entry receptor, whereas SA is the primary receptor for some species D virus and species G virus Ad52 [31,32,33,34,35]. Here, we identified Ad3, Ad35, Ad37, and Ad52 as potential candidates for virotherapy, and the receptor usage of these vectors provides important information for tumor targeting.

In the internalization assay, we measured the amount of Ad genomes in transduced cells 3 h postinfection. However, other researchers have studied even shorter incubation times such as 1 or 2 h [36,37]. We speculate that this experimental setup has no influence on the outcome of the experiments, because genome replication probably, if at all, only occurs at low levels at this time point. In future studies, different incubation times postinfection should be examined for individual Ad types to monitor genome replication. For instance, in the current study, we observed that Ad52 exhibited low infection rates 3 h postinfection, but Ad52 also resulted in the highest percentage of transgene-positive cells. Ad37 demonstrated the highest viral load 3 h postinfection, but did not exhibit the highest transgene expression levels in respective experiments. To understand the difference in infection efficiency of respective Ads, the expression of major Ad receptors by BC cell lines was analyzed (Table 1). All BC cell lines utilized in this study demonstrated low CAR (around 5% for MDA-MB-231 and SK-BR-3) or almost no CAR expression (Hs-578T and MCF7). In contrast, more than 50% of the M13SV1 cells, which served as control, were CAR positive. CD46 and DSG-2 were detected on the surface of all BC cell lines, however, some BC cell lines demonstrated only minor expression of DSG-2 (20% for DSG-2 on Hs 578T, MDA-MB-231, and MCF7).

Notably, other studies showed low levels of CAR expression in primary bladder, renal, and prostate cancers cells [38,39,40,41]. However, Martin et al. demonstrated that CAR expression is elevated in primary BC [42], and that CAR expression is positively correlated with a more undifferentiated tumor histology. Additionally, BC tissue, immunostained with CAR antibody, exhibited a more robust signal compared to other tissue types in the histopathologic sample. Moreover, Martin et al. could detect significantly elevated levels of CAR transcripts in breast tumors. Another study performed by Vindrieux et al. analyzed the link between CAR expression and estrogen signaling in BC [43]. This study revealed that CAR regulation by estrogens occurs at the transcriptional level and that the ectopic expression of CAR could increase the proliferation of BC cells. Moreover, Sakurai et al. demonstrated in a recent study that the BC cell lines MCF7 and MDA-MB-231 do express intermediate levels of CAR [44]. However, Sakurai et al. did not indicate the percentage of cells expressing CAR in their sample. Interestingly, a study conducted by Shashkova et al. observed minor CAR and CD46 receptor expression levels on BC cells compared with prostate cancer cells [16].

Cellular entry of Ad has been previously used as decisive parameter for infectivity. Hoffmann and colleagues evaluated 20 Ads types in 2 primary tumor models. In the first soft tissue sarcoma model, several Ad types, such as 35, 3, 7, 11, 9, and 22, demonstrated higher internalization efficiency than Ad5 [36]. In a second malignant melanoma model, the expression of CAR and CD46 receptors in primary melanoma was analyzed. All the immunohistochemical staining of primary cutaneous melanoma lesions from five patients indicated positive CD46 expression, whereas CAR expression could not be detected. Notably, the in situ immunohistochemistry data could be confirmed by flow cytometry analysis of the short-term cultures prepared from these melanoma lesions. Similar to the first study, some Ad types (35, 38, 3, 49, 21, 34, and 7) demonstrated higher internalization efficiency than Ad5 in melanoma cells [37]. Interestingly, the identified Ad types (e.g., Ad35) featuring highest internalization rates can also result in enhanced cancer therapy, in both in vitro and in vivo models.

To evaluate the ability of Ad to lysate BC cells, an oncolytic assay was performed. BC cell lines were infected with a dilution series of Ad types, which had demonstrated high infection and transgene expression rates in respective experiments. In concordance with results obtained in the other assays, Ad3, Ad35, and Ad52 demonstrated the most robust cell lysis efficiencies in all BC cell lines. Ad3 has a well-defined cell entry mechanism using DSG-2 and is already used in human clinical trials as oncolytic virus [45]. Ad35, which utilizes the CD46 receptor for infection, is well studied and broadly applied in gene therapeutic approaches and vaccine studies. Shashkova and colleagues studied the anticancer efficacy of Ad35, and they observed that although Ad35 had the highest cytotoxic effect in cell culture compared to other serotypes (Ad5, Ad6, and Ad11), its in vivo anticancer activity was fairly low [16]. Ad52 is yet the only discovered member of species G Ad. It is characterized by a close phylogenetic relationship with simian Ads and exhibits the lowest frequency of detection among humans [46]. With two different types of fibers, Ad52 can utilize both sialic acid-containing glycoproteins and the CAR receptor for binding to target cells [34,47]. Ad52 should be further studied to clarify the mechanisms underlying its oncolytic potency, as it may be a candidate for enhanced Ad-based BC therapy. It is of note, the Ad types used in this work are wild-type virus related Ads, in which only the early gene 3 (E3) was replaced with reporter genes (Figure 1). To apply the identified Ad types to cancer therapy, they need to be reconstructed to convert them into real oncolytic Ads. Several strategies that have been lately successfully applied for Ad5-based oncolytic Ads may be utilized to transform the wild-type-similar Ads used in this study. A very distinctive feature of oncolytic Ads is the ability to selectively replicate in tumor cells. This can be achieved by deleting viral genes that are essential for replication in normal cells, but the function can be complemented in cancer cells. For instance, E1B55K, which binds to cellular p53 and promotes G1/S transition, is an absolutely essential protein for Ad replication in normal cells while it is not needed in most tumors due to the dysfunction of the p53 pathway [48,49]. An alternative deletion can also be included in the conserved region of E1A (E1ACR2 domain). With a small 24 base pair (bp) deletion, the E1A loses its binding ability to the retinoblastoma (pRb) protein, thereby the disability of S-phase entry. Such 24-bp deleted Ads cannot replicate in normal cells, but instead only in cancer cells with deregulated cell cycle control [50,51]. Another strategy is the utilization of tumor-selective promotors, like the human telomerase reverse transcriptase (hTERT) promoter [52]. Such tumor-selective promoters can be used to control the virus replication by placing them upstream of the E1A gene. To take advantage of immunotherapy, it may also be advantageous to incorporate transgenes-expressing immune checkpoint proteins, such as PD-1 ligands, or immune-modulatory cytokine, like granulocyte-macrophage colony-stimulating factor (GM-CSF), interferon (IFN)-α, cluster of differentiation 40 ligand (CD40L), and interleukins (IL)-12 and -18 [53,54,55]. Conditional replication and immune stimulation will provide improved features to the Ads for tumor-specific oncolytic therapy.

Regarding the correlation of obtained results from different assays, we believe that multiple factors contribute to the final oncolytic effect. However, factors directly or indirectly involved in the therapeutic effect especially in the clinic remain to be discovered. If we would develop a prediction formula for the oncolytic effect, we would like to suggest the following equation: oncolysis potency = (1) viral infection + (2) transgene expression + (3) viral replication + (4) lysis of target cells. Viral infection (1) depends first on virus receptor expression, e.g., the low CAR expression on most tumors demands the exploration of alternative Ad types with CAR-independent cellular entry mechanisms. It may also be influenced by the virus cellular entry time (e.g., Ad52). Transgene expression (2) is influenced by the promoter, the expression cassette, and importantly, the type-dependent transcription and expression levels of the transgene which may be influenced by the surrounding adenoviral sequences. Note that transgene expression is of special importance if an oncolytic virus is to be armed with immune-modulatory cytokines (e.g., IFN, GM-CSF) [56,57]. Viral replication (3) can be controlled by deletion of certain viral genes or tumor-selective promotors in conditionally replicating oncolytic Ads. In general, viral replication occurs in an Ad type-dependent manner. The lysis of target cells (4) can be achieved by expressing proteins inducing necrosis and apoptosis, but can also be initiated from the lytic properties of the used virus itself [58].

In summary, the broad spectrum of naturally occurring Ads provides a great resource to identify suitable candidates for BC treatment. Of the over 20 analyzed Ad types, Ad3, Ad35, Ad37, and Ad52 demonstrated the greatest potential as innovative vectors in BC virotherapy (Table 2), which should be further modified using advanced genome engineering techniques to render these vectors tumor-cell-specific and be armed with features related to immunotherapy. These oncolytic viruses can be tested regarding their potency to kill cancer cells derived from patients in vitro or in the future, even in vivo in BC animal models.

## 4. Materials and Methods 

### 4.1. Vector Production and Titration

Recombinant adenoviral vectors expressing turbo Green Fluorescent Protein, nanoluciferase, and neomycin resistance (GLN) (Figure 1) were generated as previously described [3,59]. In detail, adenoviral genome isolated from purified adenoviruses or adenoviruses-infected propagation cells was cloned into p15A-based plasmid backbone via linear–linear homologous recombination [60]. The reporter cassette GLN was incorporated into the early transcription region 3 (E3) by linear–circular homologous recombination (LCHR) [61]. To rescue these vectors, the p15A backbone was removed with preinserted restriction enzymes; the linearized adenovirus genome containing aimed modification was transfected into HEK293 cells via calcium–phosphate transfection agent. After serial passaging to amplify the vectors to large scale, the vectors were purified by two round of cesium chloride (CsCl) gradient [62]. The physical titer of final vector particles was determined by spectrophotometry using 260 nm.

### 4.2. Cultures of Human BC Cell Lines and Human Breast Epithelial Cells

BC cell lines MDA-MB-231, Hs 578T, and MCF7 were cultured in high-glucose Dulbecco’s Modified Eagle’s Medium (DMEM, PAN-Biotech, Aidenbach, Germany); Hs 578T and MCF7 cells were supplied with 0.01 mg/mL bovine insulin. BC cell line SK-BR-3 were cultured in McCoy’s 5A Medium. M13SV1 cells (kindly provided by James Trosko, Michigan State University, East Lansing, MI, USA [63]) were cultured in McCoy’s 5A Medium and further supplemented with 10 µg/mL human recombinant EGF, 5 µg/mL human recombinant insulin, 0.5 µg/mL hydrocortisone, 4 µg/mL human transferrin, and 10 nM β-estrogen (all chemicals were purchased form Sigma-Aldrich, Taufkirchen, Germany). All the above mediums were supplemented with 10% FBS (GE Healthcare, Solingen, Germany), 100 units per mL (U/mL) penicillin, and 100 µg/mL streptomycin (PAN-Biotech, Aidenbach, Germany). All cells were maintained in a humidified atmosphere at 37 °C and 5% CO_2_.

### 4.3. Evaluation of Ad Transduction Efficiency via Luciferase Assay

The transduction efficiencies of Ads in different tumor cell lines were measured by determining reporter gene (luciferase) expression levels. Individual tumor cells were grown to confluency in 96-well tissue culture plates and infected with different viral partial numbers (vp) per cell. Precisely, 24 h after infection, luciferase activity was measured with the Nano-Glo Assay System (Promega, Mannheim, Germany), and luminescence was detected with a plate reader (Tecan, Crailsheim, Germany).

### 4.4. Analyzing Transduction Efficiencies via GFP-Positive Cells

To detect transduction efficiencies in different BC cell lines, cells were analyzed by flow cytometry. Precisely, 1 × 10^5^ cells were seeded in 24-well plates. After full confluency was reached, cells were infected at 1000 vp/c and incubated overnight. Briefly, 24 h later, cells were washed once with PBS and detached with trypsin-EDTA. Cells were resuspended in Dulbecco’s minimal essential medium containing 10% FBS, centrifuged (1500× *g*, 3 min), and washed in DPBS before they were fixed with 2% formaldehyde. Fluorescence profiles were obtained by analyzing 10,000 viable cells on Beckman Coulter Gallios Flow Cytometer. Background signal was obtained by analyzing the negative control, which was uninfected cells. The percentage of GFP-expressing cells was determined by selecting a region of fluorescence above the background of auto-fluorescence from uninfected cells.

### 4.5. Immunostaining for Receptor Detection

To detect CAR (coxsackievirus and Ad receptor) expression on the cell surface of different BC cell lines using flow cytometry, 1 × 10^5^ cells were washed with PBS supplemented with 1% BSA, centrifuged (1500× *g*, 3 min), and resuspended in 100 μL PBS/BSA and 5 μL PE-conjugated rabbit anti-hCAR antibody (Antibody Online, ABIN2649016). Following an incubation step at room temperature for 1 h, cells were washed again with PBS/BSA, to remove unbound antibodies, and resuspended in 100 μL PBS for flow cytometry using FACS (Beckman Coulter Gallios Flow Cytometer, Krefeld, Germany). As controls, each cell line without antibody was used. The PE-conjugated mouse antihuman CD46 antibody (12-0469-42, Thermo Fisher, Schwerte, Germany) was used to detect surface expression of CD46 on different cell lines. For DSG-2 detection, PE-conjugated mouse antihuman Desmoglein 2 antibody was used (CSTEM28, Thermo Fisher, Schwerte, Germany). For integrin detection, BV480 mouse anti-integrin αvβ5 (Clone ALULA, BD, Heidelberg, Germany) and BV650 mouse antihuman CD51/CD61 (Clone 23C6, BD) antibodies were used.

### 4.6. Genome Uptake Measured by Internalization Assay

To quantify the cell entry efficiency, a defined number of Ad particles (vp) was used to infect preseeded tumor cells and incubated for 3 h. Cell monolayers were digested and flushed off with trypsin, followed by extensive washing with PBS. Genomic DNA was extracted by incubation in TE buffer (10 mM Tris-HCl and 10 mM EDTA; pH 8.0) with 0.5% SDS and 0.5 mg/mL proteinase K. Subsequently, a phenol–chloroform extraction and ethanol precipitation were performed. To monitor virus genome uptake efficiency, quantitative real-time PCR (qPCR) detecting the transgene (GLN gene cassette) was performed.

### 4.7. QPCR Analysis

For quantification of the turbo Green Fluorescent Protein, nanoluciferase, and neomycin resistance (GLN) (Figure 1) reporter-tagged Ads, Primer pairs GLN-qPCR-fwd (ACC AAG CGA AAC ATC GCA TCG AG) and GLN-qPCR–rev (GCG ATA CCG TAA AGC ACG AGG AAG) binding to the transgene (GLN gene cassette) were used with a my-Budget 5× EvaGreen^®^ QPCR-Mix II reagent (Krefeld, Germany) according to the manufacturer’s protocol. PCR cycle was run and detected in the CFX Connect Real-Time PCR Detection System from Bio-Rad (Duesseldorf, Germany).

### 4.8. Oncolytic Assays with Most Promising Ad Candidates

Oncolytic assays were performed in 48 well plates. A 10-fold dilution series of individual Ads was prepared freshly to infect preseeded cancer cells. Cytopathic effect (CPE) was checked daily until at least one of the viruses on one plate at the lowest dosage showed CPE. At the latest, after 14 days, the experiment was stopped. The cells were first fixed with 3.7% formaldehyde and then, maintained adherent cells were detected by staining of attached cells with 0.5% crystal violet dye. After several washing steps, the stained plates were photographed. To quantify the survived cells, 400 µL of methanol was added to each well and incubated for 20 min at room temperature on a bench rocker with a frequency of 20 oscillations per minute. The optical density of each well was measured at 570 nm (OD570) using a Tecan plate reader [64].

### 4.9. Statistics

Statistical analyzes were conducted with Microsoft Excel. Experimental differences were evaluated by Student’s *t* test.

## 5. Conclusions

Overall, our study provides basic information on the therapeutic potential of a panel of human Ad types regarding BC virotherapy. Our finding suggests that alternative Ad types Ad3, Ad35, Ad37, and Ad52 transduce BC in a CAR-independent manner; these Ad types should be further engineered to oncolytic virus enabling tumor-specific replication and improved immunogenic response to the tumor environment.

## Figures and Tables

**Table 1:** Breast cancer (BC)-related cell lines used in this study.

Name	Histology	Characteristic	Major Adenoviral Receptors on the BC Cell Lines (Positive of All)				
						Integrin	
			CAR	CD46	DSG-2	αvβ3	αvβ5
M13SV1	Breast epithelia cells		++	+++	+++	−	+
Hs 578T	Carcinosarcoma	Triple-negative (TNBC)	−	++	+	−	−
MDA-MB 231	Invasive-ductal carcinoma	TNBC	−	+++	+	−	−
MCF7	Invasive-ductal carcinoma	Estrogen receptor positive (ER+)	−	+++	+	−	+
SK-BR-3	Invasive-ductal carcinoma	Possibly HER2/neu positive	−	++	++	+	+

**Table 2:** Summary of adenovirus infection in breast cancer-related cell lines.

Cell Line	HS 578 T	MDA-MB 231	MCF7	SK-BR-3	M13SV1
Virus uptake	Ad37, Ad3, Ad35	Ad3, Ad37	Ad3, Ad37, Ad35, Ad20	Ad37, Ad35	Ad3, Ad37, Ad5, Ad35,
Transgene expression	Ad35, Ad37, Ad52	Ad35, Ad52, Ad37, Ad50	Ad3, Ad35, Ad52	Ad35, Ad52, Ad5, Ad3	Ad5>Ad3, Ad35, Ad52
Cell lysis	Ad52	Ad5, Ad35, Ad52	Ad3, Ad35, Ad52, Ad69	Ad52, Ad3, Ad5>Ad35	Ad5, Ad35, Ad3, Ad20

## Supplementary Materials

The following are available online at https://www.mdpi.com/2072-6694/12/6/1403/s1, Figure S1: high-throughput screening of the reporter-tagged human adenovirus (Ad) library in human breast cancer (BC)-related cells with different MOIs, Figure S2: mean fluorescence intensity 1 day postinfection, Figure S3: histograms of GFP-positive cells after virus infection, Figure S4: GFP images of individual adenovirus-infected breast cancer cells, Figure S5: mean fluorescence intensity of major adenovirus receptor expression levels on BC cell lines, Figure S6: histogram of major adenovirus receptor expression levels on BC cell lines, and Figure S7: oncolytic assay using cell viability analysis.
