# Supplementary material for: Spectrum-Wide Exploration of Human Adenoviruses for Breast Cancer Therapy
Source: Cancers (Basel). 2020 May 29;12(6):1403. doi: 10.3390/cancers12061403 (PMC7352696; doi:10.3390/cancers12061403)
Supplement: Supplementary file 1 [file cancers-12-01403-s001.pdf]

cancers

MDPI

Supplementary Materials:

Spectrum-Wide Exploration of Human Adenoviruses for Breast Cancer Therapy

Nicolas Mach, Jian Gao, Lukas Schaffarczyk, Sebastian Janz, Eric Ehrke-Schulz, Thomas Dittmar, Anja Ehrhardt and Wenli Zhang

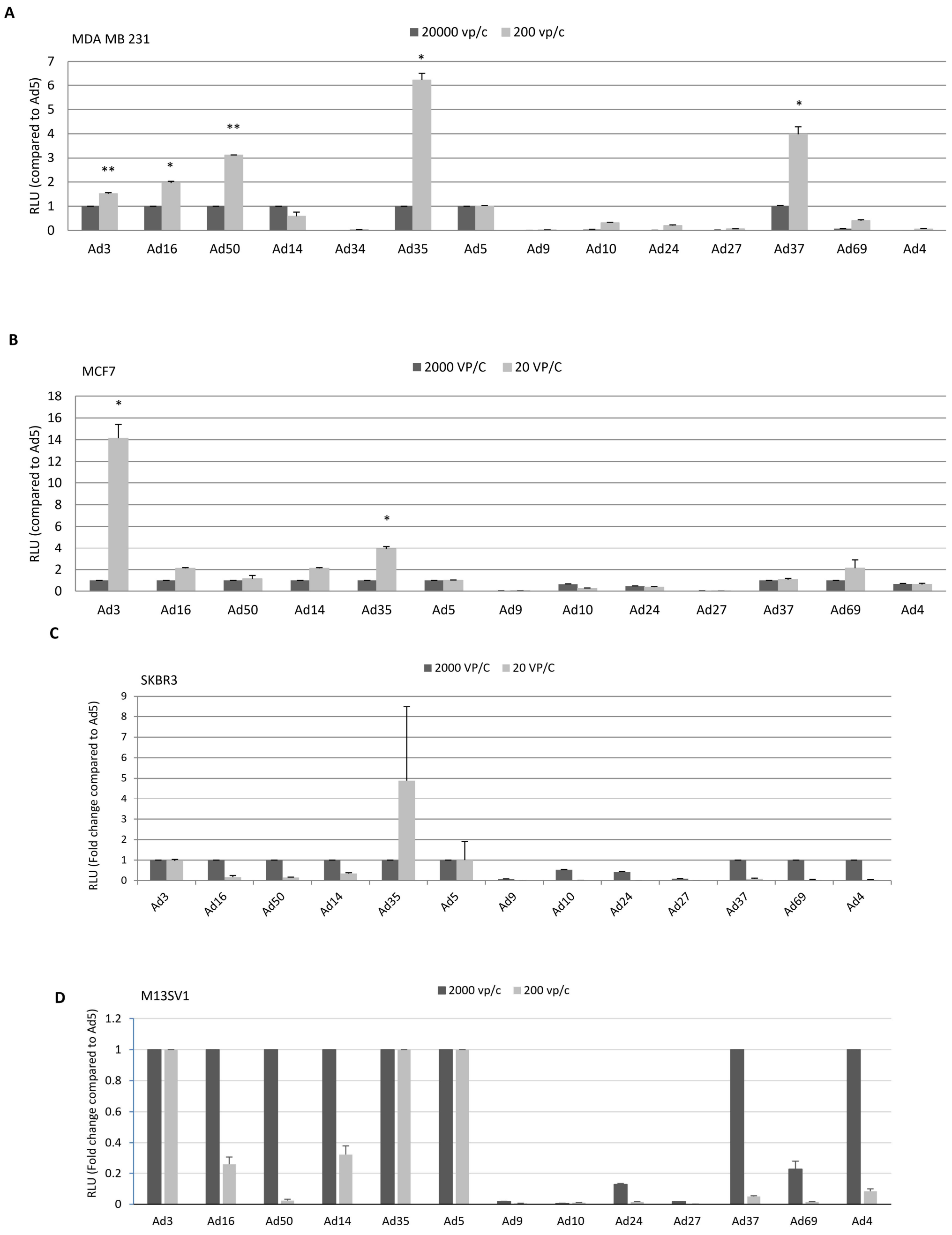

Figure S1. *Cont.*

E

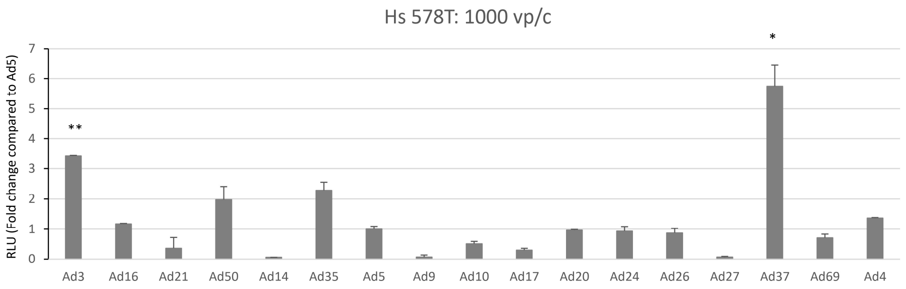

Figure S1. High-throughput screening of the reporter-tagged human adenovirus library in human breast cancer related cells with different MOIs. Transgene expression levels of different adenovirus types (Ad type number) were evaluated in breast cancer related cell lines. Cells were infected with each virus using different MOIs. Luciferase expression levels were measured 24 h post-infection by addition of Furimazine substrate and expressed as relative light units (RLU). Levels were compared to the commonly used adenovirus type 5 (Ad5) and indicated as fold change. **A**. MDA-MB-231 cells were infected with 20,000 and 200 viral particles per cell (vp/c). Most adenovirus infected cells showed saturated relative light units (RLU) at 20,000 vp/c. **B**. MCF7 cell line infected with 2,000 and 20 vp/c. Most adenovirus infected MCF7 cells showed saturated RLU at 2000 vp/c. **C.** SKBR-3 cells were infected at 2,000 and 20 vp/c. Infected cells showed saturated RLU levels for most analyzed adenoviruses at 2,000 vp/c. **D**. Breast epithelia cells M13SV1 is used as control and were infected with 2,000 and 200 vp/c. Most adenovirus Table 2. vp/c and some at 200 vp/c showed saturated RLU. **E**. Hs 578T cells were infected at 1,000 vp/c. Error bars, ±SEM of three independent experiments. * $p < 0.05$; ** $p < 0.01$; compared to Ad5 control.

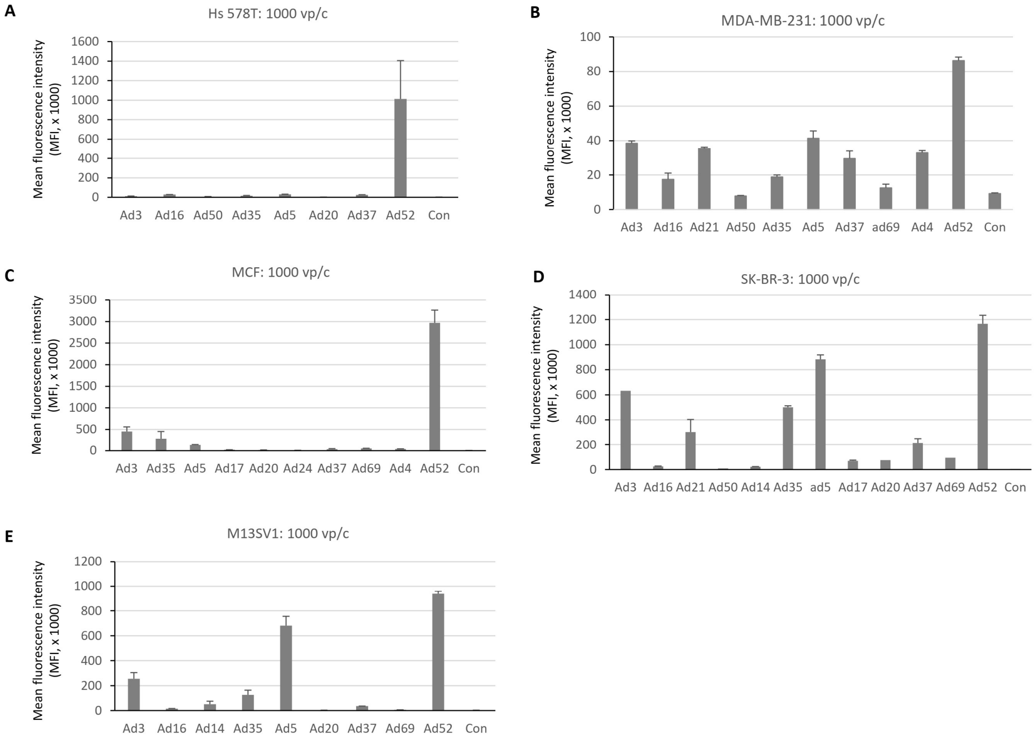

Figure S2. Mean fluorescence intensity 1 day post-infection. Cells were infected with 10 different Ads at 1,000 viral particles per cell (vp/c). GFP expression levels were analyzed 24 h post-infection by FACS analyses. Uninfected cells (negative controls) were used to set the background gate below 1%. A total of 10,000 viable cells were counted. **A–D**. Breast cancer originated tumour cell lines. **E**. Breast epithelia cell line M13SV1 was used as control. Error bars represent mean ± SD ($n = 2$).

A. Hs 578T-GFP

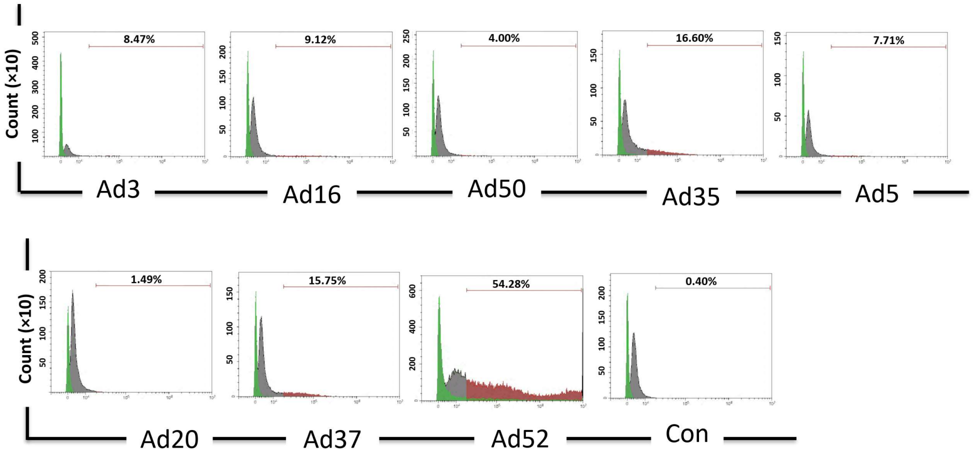

B. MDA-MB-231-GFP

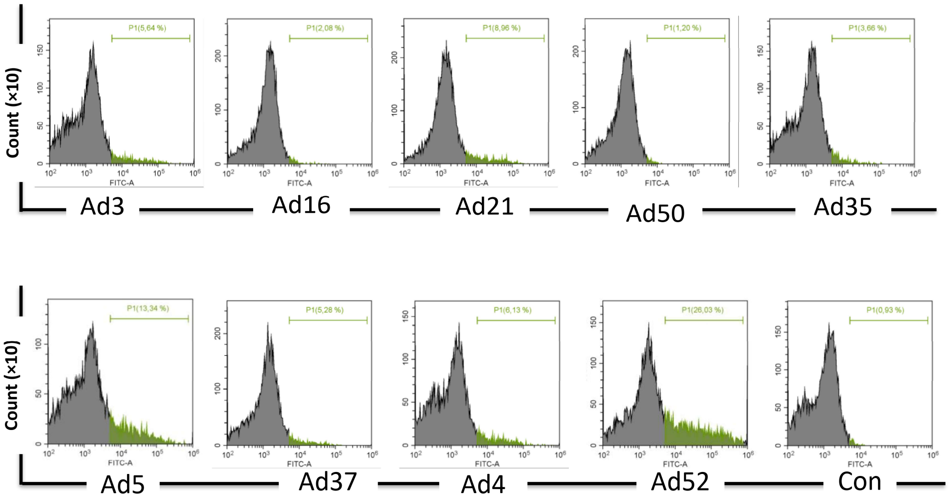

C. MCF7-GFP

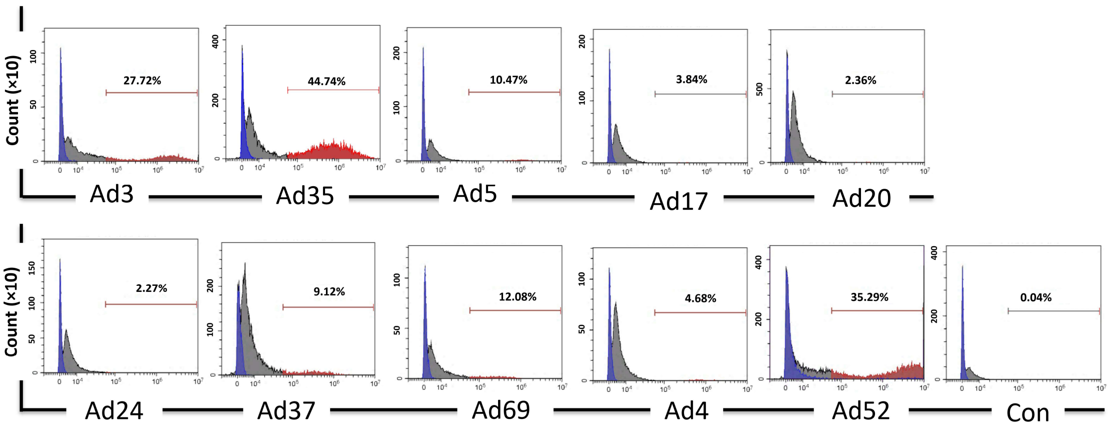

Figure S3. *Cont.*

D. SK-BR-3-GFP

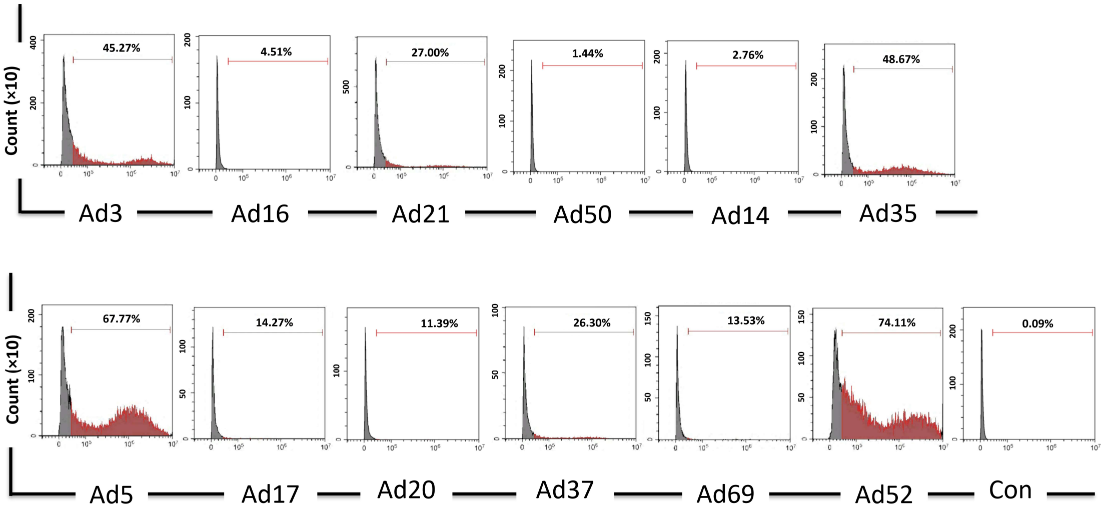

E. M13SV1-GFP

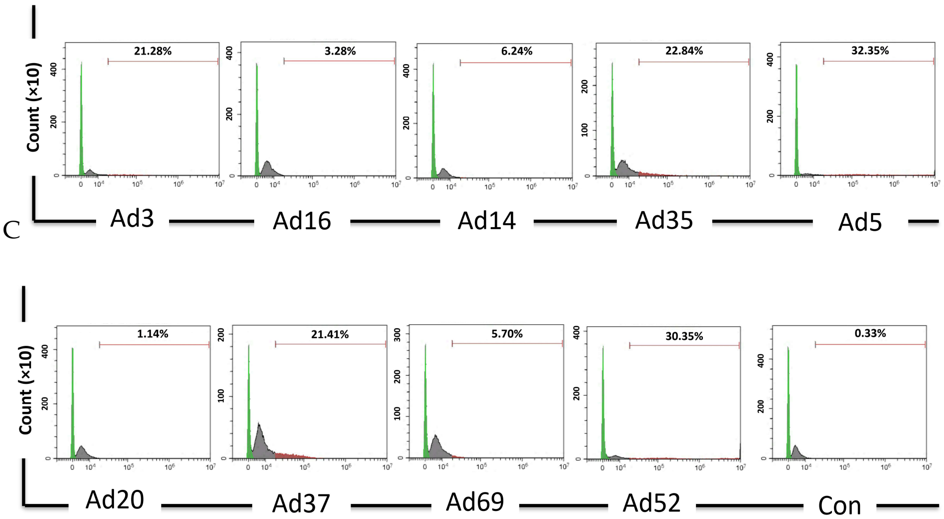

Figure S3. Histograms of GFP positive cells after virus infection. Cells were infected with ~10 Ads at 1,000 viral particles per cell (vp/c) and GFP expression levels were analyzed 24 h post-infection by flow cytometry analyses. Uninfected cells (negative controls) were used to set the background gate below 1%. Percentage provided indicates % of GFP-positive cells. A total of 10,000 viable cells were counted. **A**–**D**. BC originated tumor cell lines. **E**. Breast epithelia cell line M13SV1 were used as control. Error bars represent mean ± SD (n = 2).

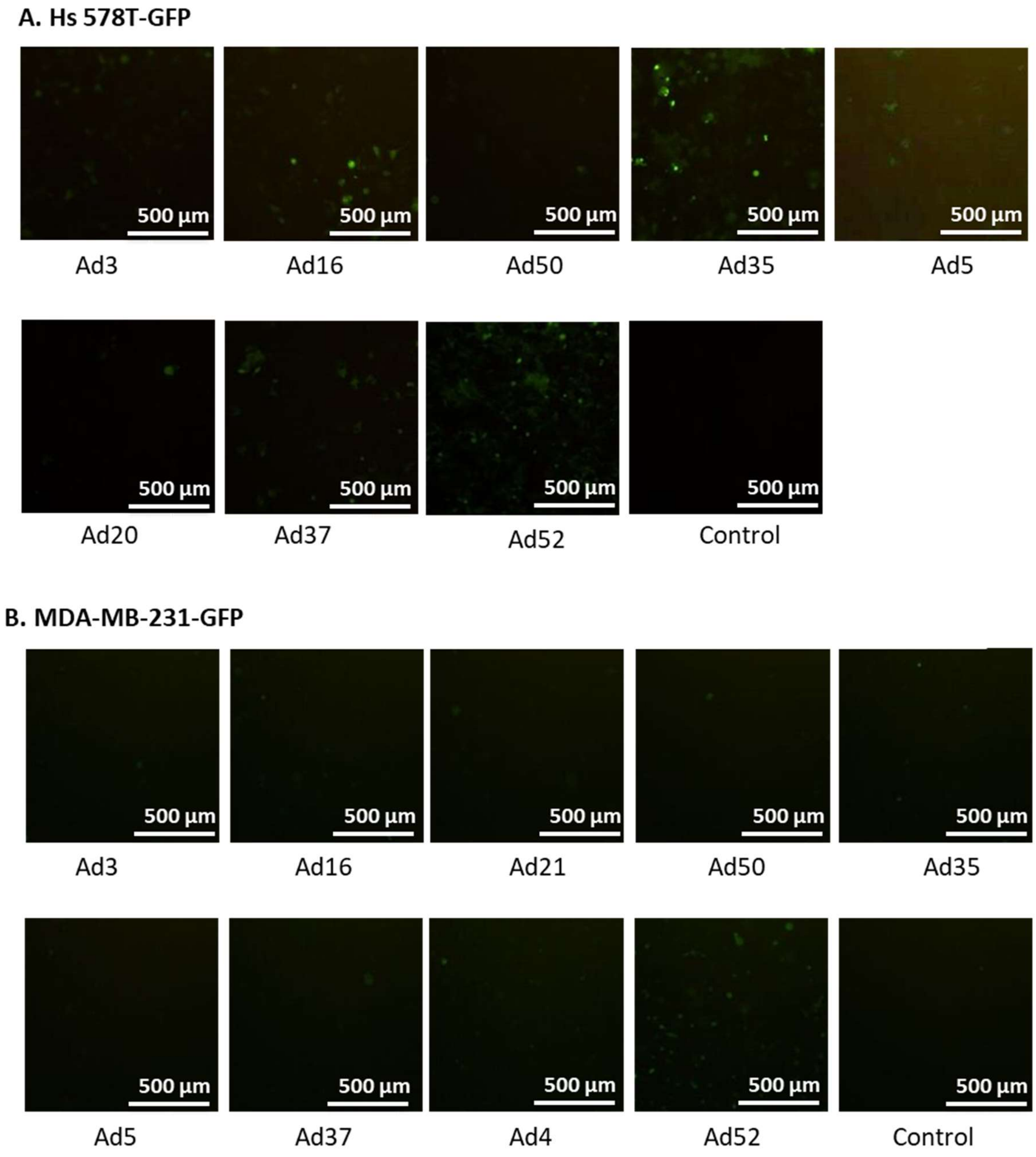

Figure S4. *Cont.*

C. MCF7-GFP

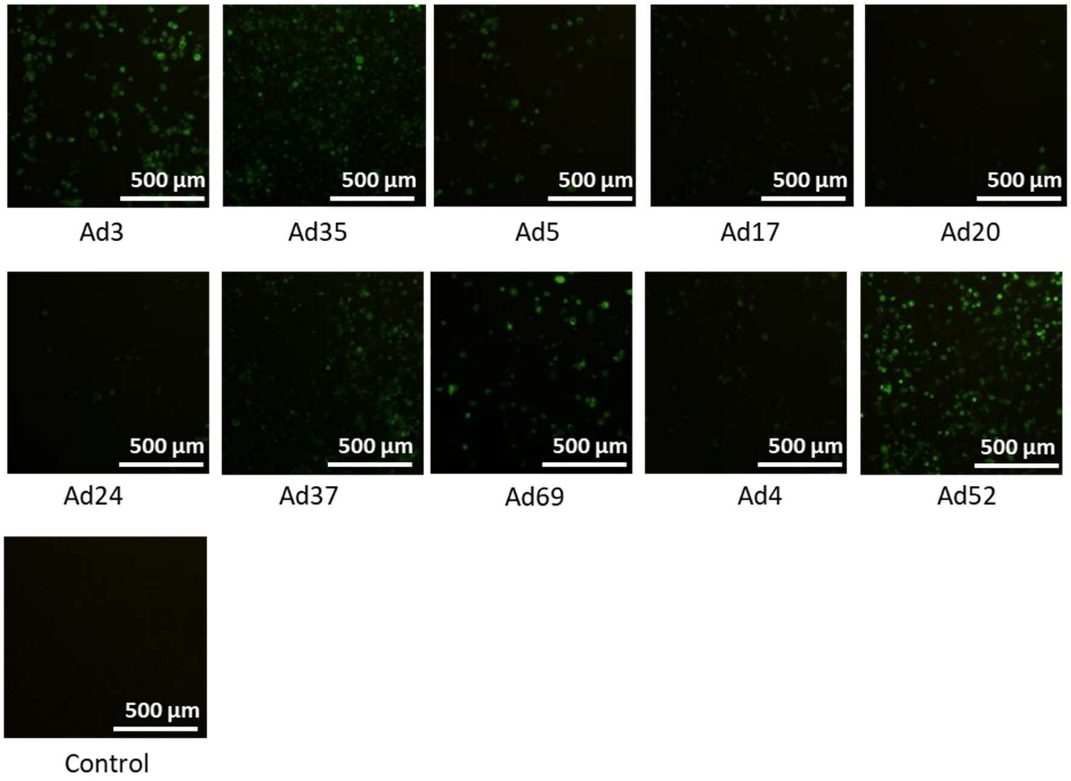

D. SK-BR-3-GFP

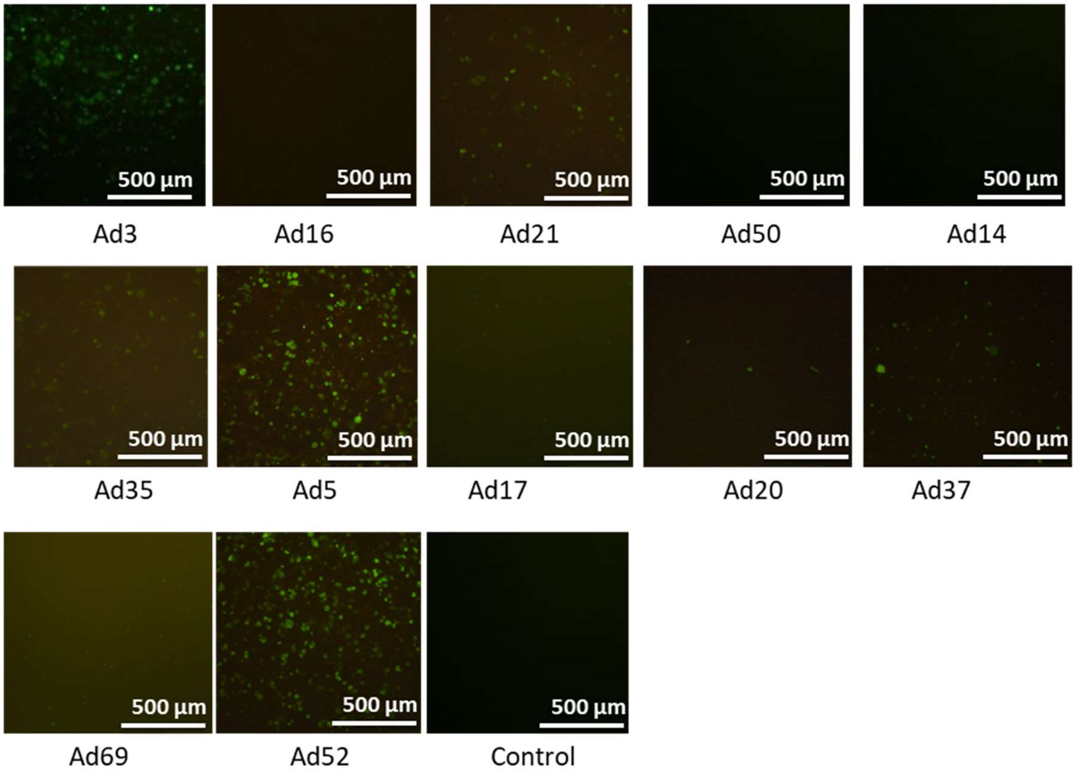

Figure S4. *Cont.*

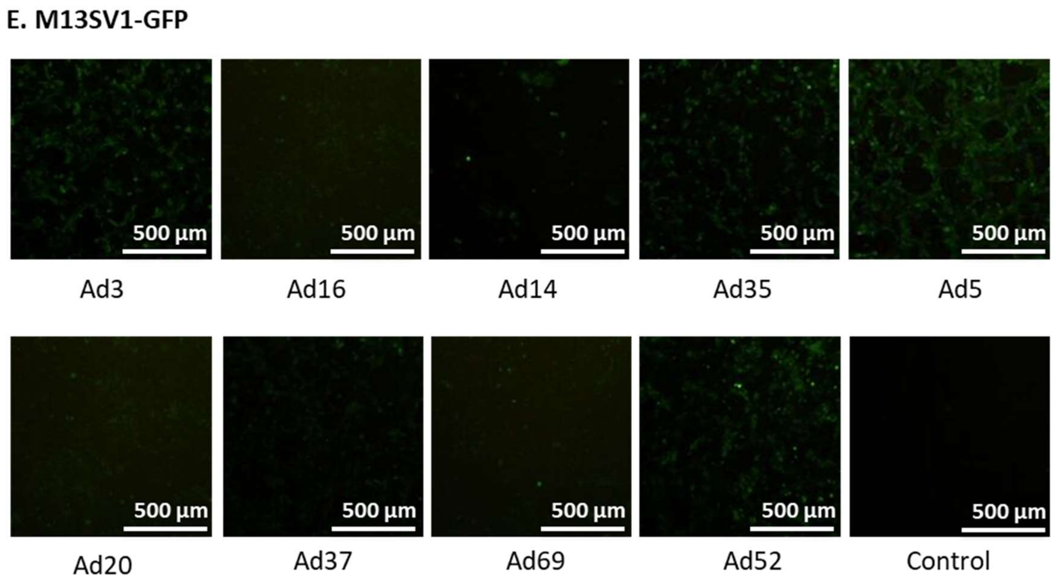

Figure S4. GFP images of individual adenovirus-infected breast cancer cells. Cells were infected with respective adenoviruses at 1,000 viral particles per cell (vp/c) and GFP expression levels were analyzed 24 h post-infection. Uninfected cells were used as negative controls (control). **A–D**. Breast cancer originated tumor cell lines. **E**. Breast epithelia cell line M13SV1 was used as control.

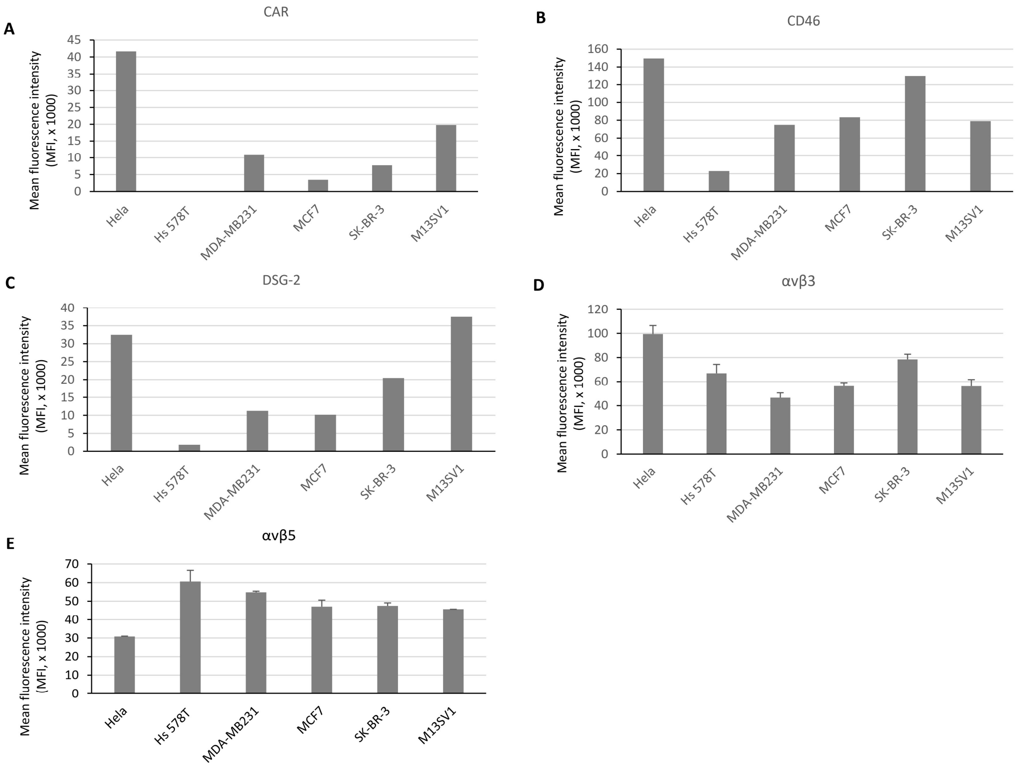

Figure S5. Mean fluorescence intensity of major adenovirus receptor expression levels on BC cell lines. Cells were stained with PE-labeled antibodies against CAR (**A**), CD46 (**B**), DSG-2 (**C**) and integrins (**D**,**E**). Receptor expression was evaluated via flow cytometry. Hela cells were used as positive control. Unlabeled cells (negative controls) were used to set the background gate below 1%. A total of 10,000 viable cells were counted.

A. CAR-PE

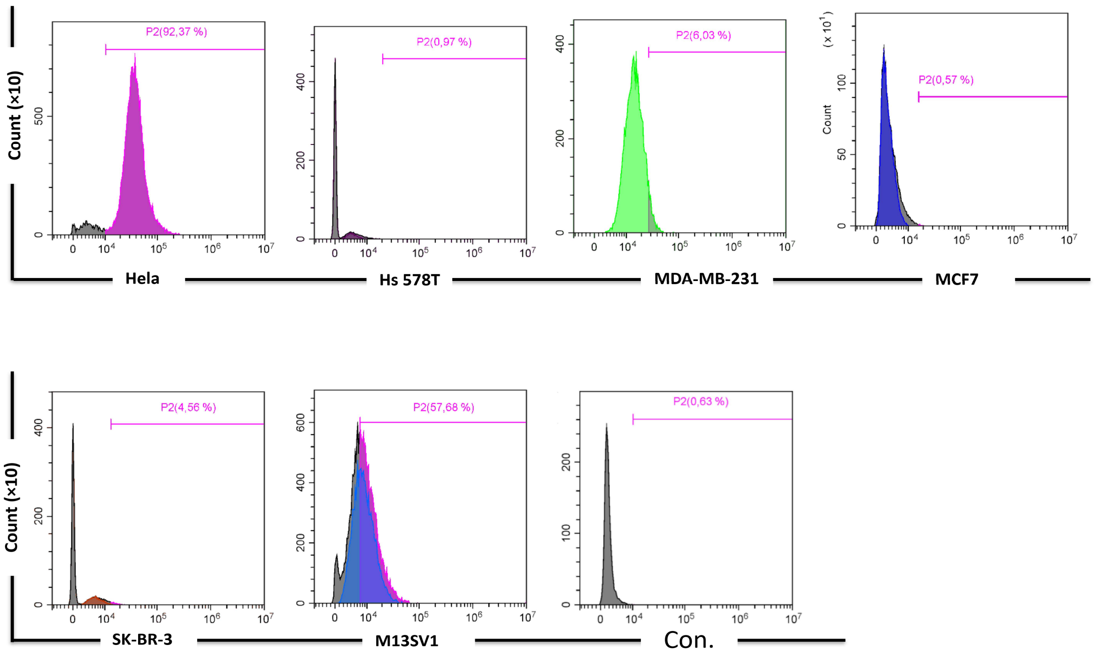

B. CD46-PE

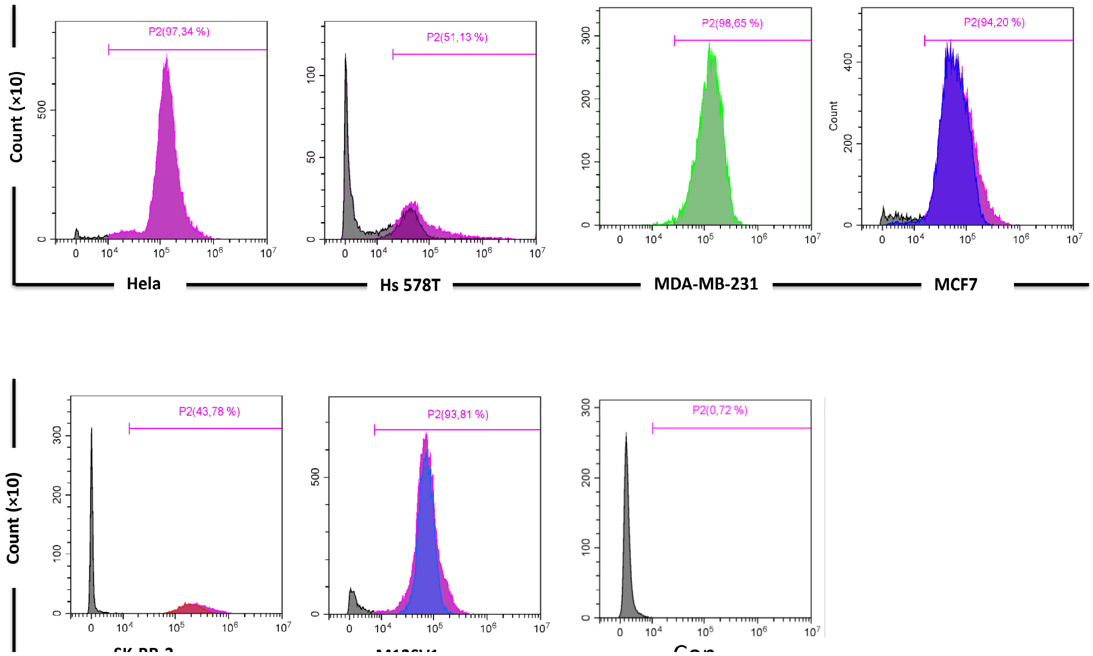

Figure S6. *Cont.*

C. DSG-2-PE

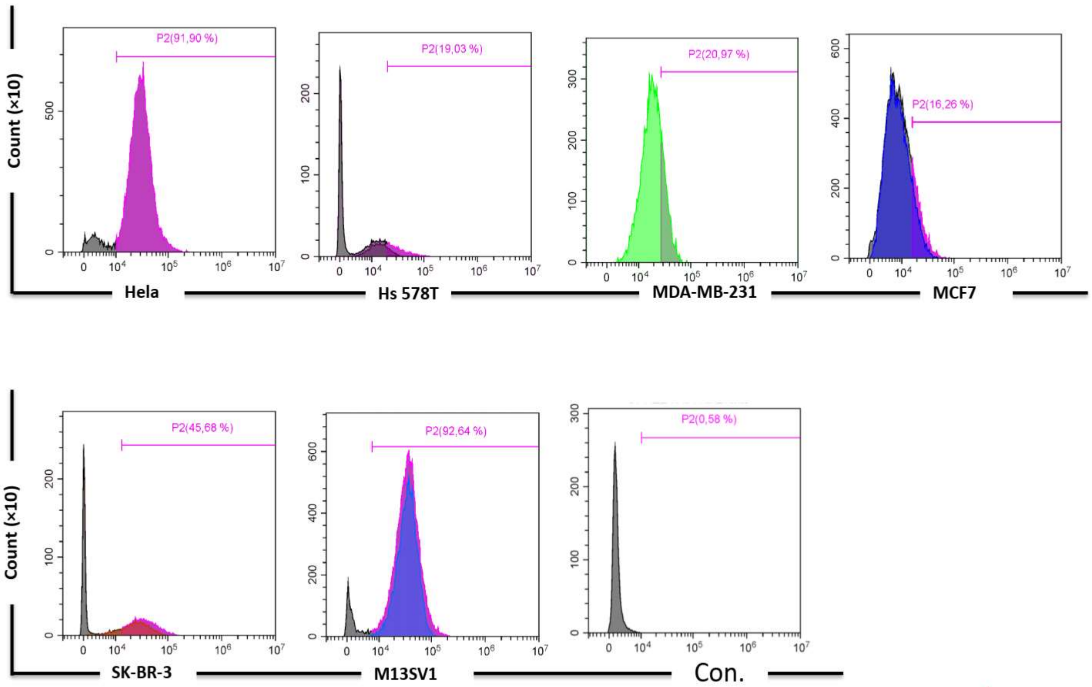

D. αvβ3-BV650

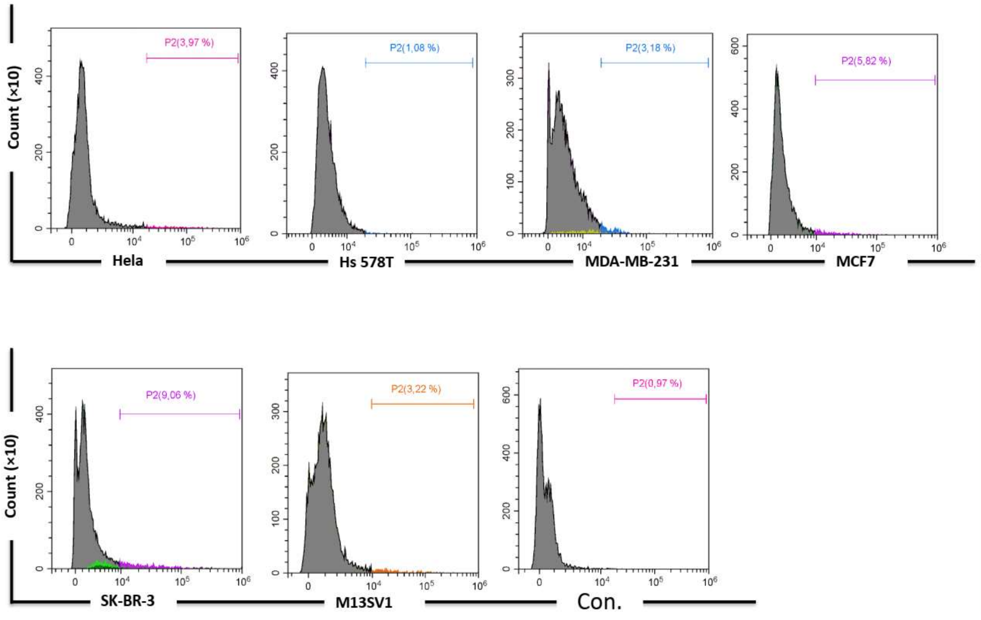

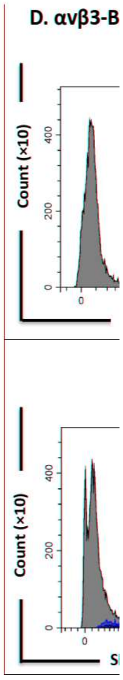

Figure S6. *Cont.*

E. αvβ5-BV480

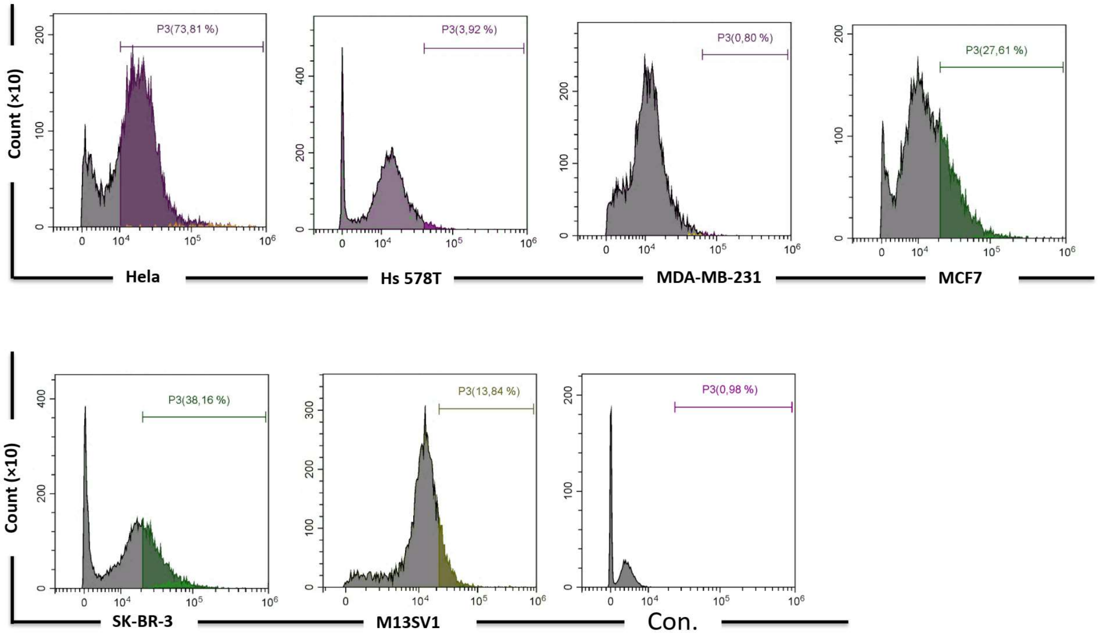

Figure S6. Histogram of major adenovirus receptor expression levels on BC cell lines. Cells were stained with PElabeled antibody against CAR (**A**), CD46 (**B**), DSG-2 (**C**) and integrins (**D**,**E**). Receptor expression was evaluated via flow cytometry. Hela cells were used as positive control. Unlabeled cells (negative controls) were used to set the background gate below 1%. A total of 10,000 viable cells were counted.

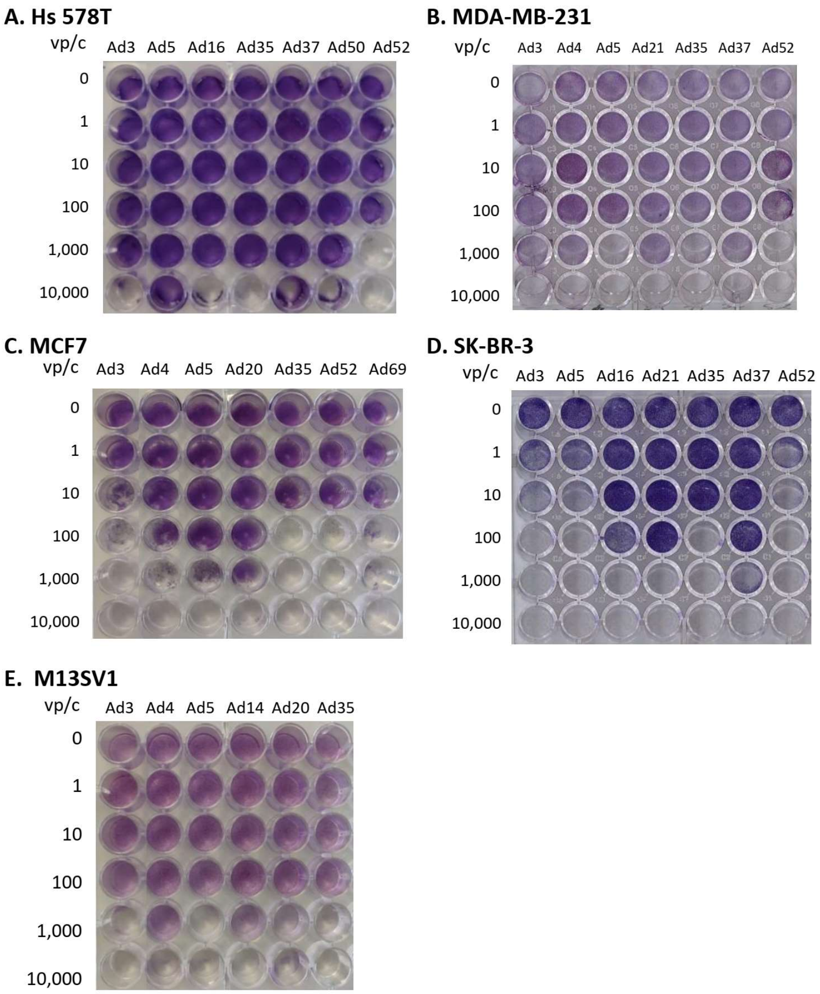

Figure S7. Oncolytic assay via cell viability analysis. Cells were infected with selected viruses at various ratios (0, 1, 10, 100, 1,000 and 10,000 virus particles per cell (vp/c)). Crystal violet staining of viable cells was used to evaluate oncolytic activity.
